# Protecting SOME/IP Communication via Authentication Ticket

**DOI:** 10.3390/s23146293

**Published:** 2023-07-11

**Authors:** Seulhui Lee, Wonsuk Choi, Dong Hoon Lee

**Affiliations:** School of Cybersecurity, Korea University, Seoul 02841, Republic of Korea; hoisulhi@korea.ac.kr (S.L.); beb0396@korea.ac.kr (W.C.)

**Keywords:** SOME/IP, in-vehicle network, automotive Ethernet, security, authentication ticket

## Abstract

Designed using vehicle requirements, Scalable service-Oriented MiddlewarE over IP (SOME/IP) has been adopted and used as one of the Ethernet communication standard protocols in the AUTomotive Open System Architecture (AUTOSAR). However, SOME/IP was designed without considering security, and its vulnerabilities have been demonstrated through research. In this paper, we propose a SOME/IP communication protection method using an authentication server (AS) and tickets to mitigate the infamous SOME/IP man-in-the-middle (MITM) attack. Reliable communication between the service-providing node and the node using SOME/IP communication is possible through the ticket issued from the authentication server. This method is relatively light in operation at each node, has good scalability for changes such as node addition, guarantees freshness, and provides interoperability with the existing SOME/IP protocol.

## 1. Introduction

A vehicle comprises many electronic control units (ECUs) developed by various companies. Each electronic control unit is responsible for its dedicated functions, such as engine control, brake control, steering control, infotainment system, etc. When necessary, ECUs communicate with each other through the network. In recent years, the requirements for software installed in vehicles have become much more advanced based on functions such as advanced driver assistance systems (ADAS), infotainment, and over-the-air (OTA) software updates. Following this trend, software complexity has risen accordingly, and either vulnerabilities or the number of attack surfaces of the entire system could increase. In this situation, standardization and development of automotive software frameworks like the standardized automotive software reference architecture AUTOSAR have many advantages, including reducing the complexity of software installed in vehicles, maintaining software quality, and reducing research and development costs.

In the AUTOSAR platform, high-bandwidth Ethernet communication is utilized to enable seamless communication between various functionalities within a vehicle, requiring fast processing of large amounts of data. (However, because bus communication like the Controller Area Network (CAN) protocol has specific advantages, it is still used for control messages where real time and reliability are essential). To this end, AUTOSAR adopted Scalable service-Oriented MiddlewarE over IP (SOME/IP) as an Ethernet middleware protocol [1,2,3,4,5,6]. SOME/IP is designed for service-oriented middleware (SOM) based on automotive requirements and is used to transmit vehicle control messages between multiple electronic control devices. SOME/IP is flexible and scalable, supporting various communication methods and data types. In addition, it has many other advantages, like not depending on the platform and being economical because it transmits data only when the receiver needs the data. Since the data exchanged through SOME/IP in the vehicle are mainly vehicle control messages, it cannot be emphasized enough how vital SOME/IP communication security is. A vehicle control message that has been maliciously manipulated by an attacker causes the vehicle’s control system to operate in a way the driver does not want, which can lead to life-threatening consequences for the driver and passengers. It does not happen only in movies or the imagination, as demonstrated in the Fiat Chrysler remote hacking demonstration by Charlie Miller and Chris Valasec [7,8]. There are also well-known attacks and studies in the automotive industry [9,10], not only through wired communication, but also through wireless, including but not limited to intercepting and replaying keyless entry system signals, compromising control systems for unauthorized manipulation, and exploiting GPS systems for tracking or falsifying vehicle location data. Therefore, the security of SOME/IP is considered essential and is a task that must be solved.

Man-in-the-middle (MITM) attacks that can be applied to SOME/IP as well as relevant mitigation methods have been introduced in other studies. We considered these attacks and the limitations of other studies. The main contributions of the presented scheme in this paper are:

1. Protection against MITM attacks by allowing only authenticated end nodes to communicate using an authentication ticket released by an authentication server
2. Efficient operations for secure communication via a symmetric key
3. High portability for security expansion when the current SOME/IP protocol is used
4. Scalability of node changes by reducing the number of required update points
5. Prevention of replay attacks by verifying the freshness token

Section 2 summarizes the primary contents and characteristics of SOME/IP, and Section 3 reviews related works. Section 4 details the proposed scheme, and Section 5 analyzes its security, including ProVerif. The proposed scheme is evaluated in Section 6, and the conclusion of this study is presented in Section 7.

## 2. SOME/IP

### 2.1. SOME/IP

**Figure 1:**
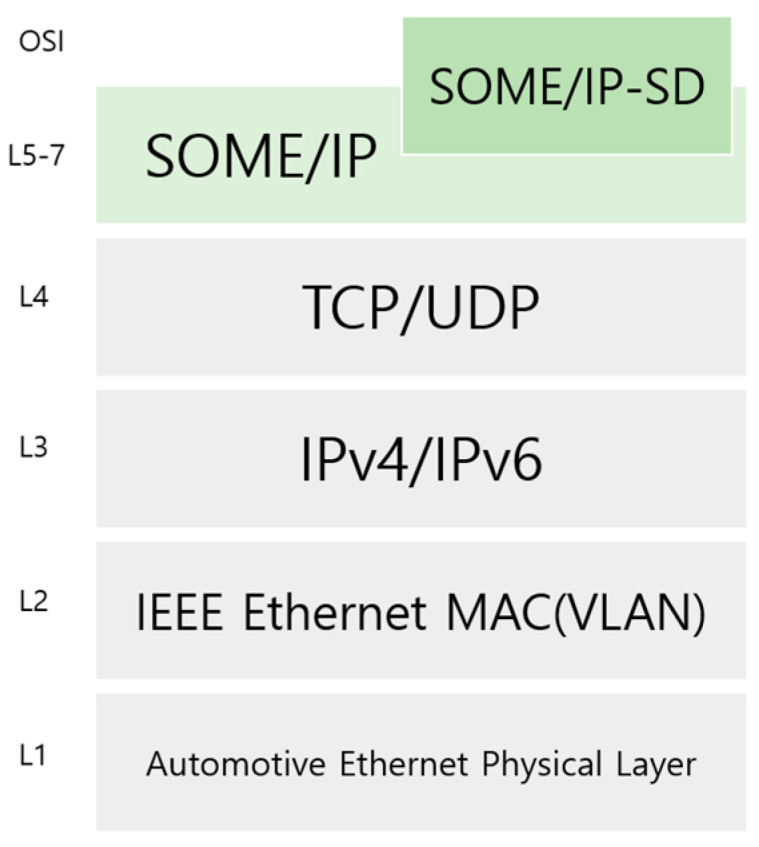
SOME/IP based on OSI 7 layer.

First, we overview SOME/IP and how it works. SOME/IP is an automotive Ethernet middleware communication protocol optimized to transmit vehicle control messages of various sizes and formats on different operating systems for each device. As shown in Figure 1, SOME/IP is implemented in OSI Layer 5–7 and has the advantages of being a protocol optimized for vehicle requirements and being readily applicable to various platforms and limited-resource environments. AUTOSAR has designated SOME/IP as an automotive Ethernet communication standard middleware. While traditional vehicle CAN communication is static, SOME/IP communication is dynamic and service-oriented because it is designed based on service-oriented architecture (SOA). SOME/IP is capable of various types of communication, such as publish/subscribe, fire/forget, notification of specific events or subscription, and general request/response communication. Only the client that needs a particular service can receive data by requesting a service.

### 2.2. SOME/IP-SD

The OfferService message is multicast or unicast to the clients to deliver information about the service provision. If the client does not receive the required OfferService message within the valid time, it sends a FindService message to find its provider. These messages are SOME/IP-SD (Service Discovery) [11]. SOME/IP-SD enables flexible communication of SOME/IP, such as searching for and subscribing to necessary services. Through SOME/IP-SD, only clients who need data for a specific service can receive data to communicate economically. Economic communication refers to the practice of not transmitting unnecessary data. However, even though SOME/IP-SD messages are mostly multicast, there is no way to trust the endpoint because there is no separate verification method for the transmitted endpoint. The advantage of flexible communication of SOME/IP, though, can be a security vulnerability. As a result, researchers have revealed several feasible MITM attacks related to SOME/IP communication [12,13]. Since the importance of protecting vehicle control and messages is very high, a secure SOME/IP communication method that is both robust against possible attacks while maintaining the advantages of the SOME/IP-SD protocol is required.

### 2.3. SOME/IP MITM Attack

SOME/IP-SD makes flexible service provision and searches possible. It is no exaggeration to say that a feasible MITM attack related to SOME/IP originates from information sharing on services transmitted through multicasted SOME/IP-SD messages. If an attacker can come within the range of a network that is capable of receiving multicast messages, or if they succeed in infecting a normal node, the attacker can easily find information about which instance provides a particular endpoint for a specific service. When SOME/IP-SD messages are transmitted and received, there is no procedure or policy that can verify consistency between each endpoint and service, making various attacks possible.

**Figure 2:**
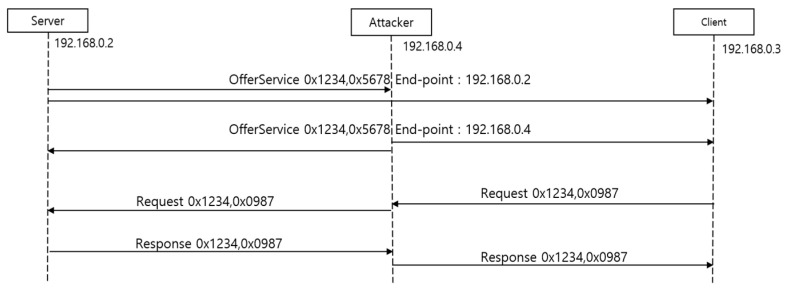
A representative SOME/IP MITM attack.

Figure 2 shows a representative SOME/IP MITM attack [12]. If a server (192.168.0.2) sends an OfferService message for its service (the service ID is 0x1234, and the instance ID is 0x5678) by multicast, both a legitimate client (192.168.0.3) and an attacker (192.168.0.4), who has successfully invaded an in-vehicle network, can receive the message. The attacker changes the message’s endpoint to their own and re-sends it via multicast. Now, the client wants to use the service and sends a request. Depending on the SOME/IP implementation, the client can request the service to the first or last offered endpoint. Suppose the client sends it to the last provided endpoint, that is, the client sends a Request message to the attacker. Then, the attacker forwards it by changing its endpoint to the attacker’s. When a server receives a Request message from an attacker, it sends a Response message, and then the attacker can read and change the data as they want and re-send the message to the client. In this way, an MITM attack can occur. A detailed description of representative MITM attacks is explained in the blog [12] written by Shir Mousseri from ARGUS cyber security. Zelle et al. [13] present various MITM attack scenarios that can occur in SOME/IP, as well as the feasibility of the attacks in detail. Since actual vehicle control messages can create hazardous situations when attacked, a method that mitigates MITM attacks and does not affect the vehicle’s requirements is needed. Several proposals have been published as a result of these studies.

## 3. Related Works

To ensure the secure usage of SOME/IP, there are various papers [13,14,15,16,17] available. Among them, I have compared two early papers with my own research.

### 3.1. Secure SOME/IP

Among the papers suggesting methods that mitigate SOME/IP MITM attacks, Iorio et al. [14,15] presented an additional handshake before actual SOME/IP communication between the server using SOME/IP communication and all clients with a specific service. This SOME/IP communication protocol introduced a process to establish each session and subsequently was configured to proceed. The tasks carried out in the added handshake are as follows:

1. Mutual node authentication through the public key infrastructure (PKI) method
2. Policy check among nodes for the service listed in the certificates (e.g., a service providing availability or service subscription availability)
3. Sharing the SOME/IP communication security level to be used
4. Sharing the security material (e.g., key value, etc.) for SOME/IP communication

For this to be possible, each node’s private key and certificate had to be configured first in all ECUs in advance. The security level would be divided into three levels according to the security level exchanged during the handshake process. One level was “nosec”, which was the same as the existing SOME/IP message and would mean no action was taken on the actual SOME/IP message after the handshake. Another was “integrity”, which provided only message integrity, and the last was “confidentiality”, which guaranteed message confidentiality. In the handshake process, one message was exchanged from Client to Server, and Server to Client. Compared to the SOME/IP protocol, two message transmissions would be added to authenticate the node and exchange security materials for secure communication from that point.

In this existing method, the server providing the service had to conduct a 1:1 handshake with each client and maintain the session. Therefore, even a node that did not need to subscribe to a service or request data would immediately have to maintain a session by performing a handshake. From the viewpoint of economical communication, which was the advantage of the SOME/IP protocol, this was not ideal. In addition, since an asymmetric key-based operation was involved, the protocol performed relatively heavy operations, so at times it would be difficult to smoothly process the operations for nodes with limited resources. In addition, a replay attack could be possible here because secure SOME/IP communication neither suggested a re-keying mechanism for a long-running service, nor did it include processing for message freshness.

### 3.2. SESO-RC, SESO-AS

Zelle et al. [13] presented several SOME/IP MITM attack scenarios and showed the feasibility of actual MITM attacks using the revealed attack procedures. The authors suggested two security extensions as MITM attack mitigation methods. SESO-RC, the first proposed method, adopted a PKI-based mutual authentication method similar to the secure SOME/IP [14,15] in Section 3.1, but unlike the latter, it did not have an additional handshake process. In the message exchange process using the SOME/IP protocol, the DH key exchange was applied to create a session key. Also, SESO-RC was not a fine-grained policy, as it had keys and policies per ECU. Having per-end node policies is preferred because multiple SOME/IP services can be provided in one ECU. Similarly to secure SOME/IP, SESO-RC also used asymmetric key operations. Because of this, a security extension operation could be restricted if a process operated on an ECU with limited resources. The policies among the nodes would be specified in the certificate in both secure SOME/IP [14,15] and SESO-RC [13]. After verifying certificates, the nodes would check service provision/subscription availability based on the policies written in the received certificate. Note that this method is inflexible to node change. This means that, if a new node providing a SOME/IP service were added to a vehicle through a software upgrade and existing nodes wanted to use the new service, they would have had to adopt all relevant ECU software updates since the certificates needed to be re-issued to have a new policy. Therefore, changing a node or service would entail a lot of additional overhead, such as revoking and managing the existing certificate and updating the related ECU’s SW to obtain a certificate.

In SESO-AS, the second security extension proposed in the same paper [13], an additional authentication server was introduced. SESO-AS assumed that the symmetric key and policy for each ECU were set in the authentication server in advance. When the server transmitted the OfferService message, including the session key, to the authentication server with the message MAC value [18], the authentication server would first verify the MAC with the pre-set server key and then check the policy. Afterward, the authentication server played a role in distributing OfferService messages and a session key to ECUs that were able to subscribe to this service. In this process, the session key was encrypted with the pre-configured long-term key for each ECU receiving OfferService messages. A so-called “container” with the encrypted session key and MAC were created per receiving ECU. The authentication server would configure a new data structure called an OS, which included containers, and multicast it to receiving clients. From the perspective of one client ECU, unnecessary data would have been received because containers for other ECUs must first be obtained. From the standpoint of the authentication server, unnecessary containers must be created even for clients not presently subscribed, assuming they had permission. For these reasons, SESO-AS is inefficient in many respects.

### 3.3. Comparison

Table 1 shows the comparison among various schemes introduced so far in this paper. “Scalability” means that a scheme experiences a few changes when integrating a new node. We can know whether a scheme’s policies and keys are fine-grained through “Granularity”. “Overhead” tells how much cryptographic operational overhead occurs when the scheme is applied, and we can know whether or not a scheme is compatible with the current SOME/IP protocol by looking at “Compatibility”. Finally, “Efficiency” shows if the required additional operation is applied only for a node that needs it. Ticket-based SOME/IP indicates the novel scheme we are proposing in this paper. Ticket-based SOME/IP and SESO-AS support high scalability thanks to the leveraging of an additional server. As ticket-based SOME/IP and Secure SOME/IP manage keys and policies per node; they are fine-grained granularity. For the overhead aspect, Secure SOME/IP and SESO-RC have high overhead because of public key cryptography operation. Ticket-based SOME/IP and Secure SOME/IP can select whether a security expansion applies or not per service, which assures compatibility with the current SOME/IP protocol. Secure SOME/IP makes a 1:1 connection with every node if it can use SOME/IP. SESO-AS’s authentication server makes containers for every node that can use the service. For these reasons, “Efficiency” is marked as “Low” for Secure SOME/IP and SESO-AS.

We found that “Granularity” is crucial because if policies and keys are not fine-grained, SOME/IP MITM and other attacks can occur. This is because, if an attacker can compromise one node, they can disguise all nodes in the same ECU since they share keys and policies. Therefore, we evaluated our scheme only with Secure SOME/IP.

Considering these points, we suggest a flexible scheme that expands with a minimum SW update when changing nodes, mutually trusts the server and client, selectively performs only services that require additional security expansion, and minimizes overhead for each node.

## 4. Proposed Scheme

### 4.1. Prerequisites

Our proposal includes SOME/IP communication that can be operated by checking the services and node consistency through mutual server and client authentication as well as service policy verification [19] via an authentication server (AS) and authentication tickets issued from the server. The basic concept of this method is similar to Kerberos [20], but it is applied to the existing SOME/IP protocol and configured in a more simplified way in consideration of performance. The node mentioned in this paper refers to the end node process that provides and uses actual services. The node could be a functional cluster or application. Before describing our proposed method, we first outline the limitations:

(1) All SOME/IP end nodes have a cryptographically secure long-term key, and policies are defined for service provision/availability between all endpoints. Keys and policies for each node must be provisioned in advance in an AS and in a secure manner.

(2) Time sync must be ensured among all ECUs in the vehicle.

(3) Since both node-to-node authentication and policy verification are performed in the AS, it is assumed that the AS is trustworthy. A system (including the key, policy, etc.) must be safely managed through various security solutions such as secure boot and hardware security module, and an SW update must also be controlled through reliable methods. Note that the detailed techniques for this are outside the scope of this paper and will not be discussed in detail.

(4) We assume that the database for keys and policies managed by the AS is configured in a way that allows secure and fast operation according to the situation of each server. Note that database management and optimization are outside the scope of this paper and will not be discussed in detail.

### 4.2. Attack Models

The attack models considered for this paper are listed below. These attack models are well-known attacks that can be launched against the in-vehicle network, and they are attacks that could cause serious consequences if they occurred during the delivery of a vehicle control message:

(1) Man-in-the-middle attack: An attacker can intercept an original message and send a modified one.

(2) Replay attack: An attacker can gain unauthorized access or cause a malfunction by reading, recording, and replaying legitimate messages transmitted between two nodes.

(3) DoS attack: An attacker can prevent a client node from operating normally by replaying the Response message.

(4) Spoofing attack: An attacker can send and receive messages illegitimately by pretending to be a legitimate device in the in-vehicle network.

### 4.3. Overall Flow

**Figure 3:**
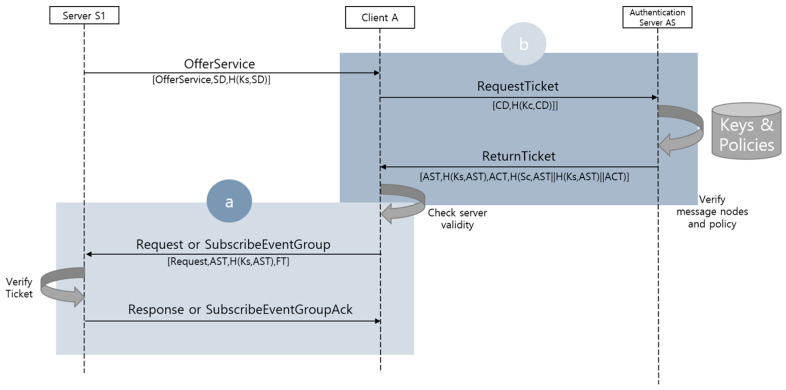
Overall SOME/IP communication flow for proposed scheme ((**a**) original flow, (**b**) additional flow).

The schematic flow of the method proposed in this paper is shown in Figure 3 below. 

In general SOME/IP communication, the Server S1 multicasts/unicasts OfferService messages to clients. In the proposed method, the OfferService message is transmitted with a flag that indicates that the service requires an authentication ticket corresponding to the importance of the provided service. Assume that Client A receives the OfferService message and wants to subscribe to this service. First, our method verifies whether the corresponding service requires a ticket. If it is not needed, it immediately responds in the original communication method (e.g., Request message). This is shown in process “a” of Figure 3. After this, our method additionally performs process “b” to obtain an authentication ticket if necessary. The formulas under the directed edge for each step represent the rough composition of added data when a ticket is required. Table 2 shows the used notation in the figure and corresponding detailed descriptions, which can be found starting in Section 4.4 of this paper. In process “b”, two messages are added: a RequestTicket message, which requests an authentication ticket from the AS, and a ReturnTicket message, which returns the requested authentication ticket. The proposed scheme consists of four phases: OfferService, RequestTicket, ResponseTicket, and Request.

### 4.4. OfferService Phase

**Figure 4:**
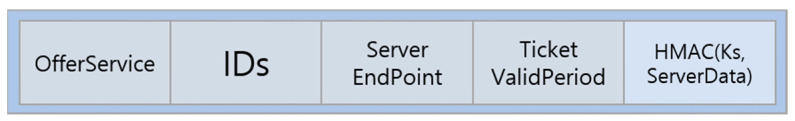
Data added behind an OfferService message when a service requires a ticket.

This message is sent as a multicast or unicast message to notify the client that the server provides a specific service. In this phase, the server can set a flag to indicate whether or not an authentication ticket is required to use the service, which can be selectively applied depending on the importance of the service. In actual implementation, this flag is set using unused bits in the message types of SOME/IP messages. When creating an OfferService message that requests a ticket for the service in the server, ServerData is additionally configured, as shown in Figure 4 below, after the original OfferService message payload part. For a detailed description of the notation used in Figure 4, refer to Table 3.

When a ticket is required, the parts that need to be added/changed in the existing OfferService message are the setting of a flag bit indicating that the service requires a ticket, the configuration of ServerData, and the additional HMAC (hash-based message authentication code) value created with a long-term server key. By sending the identifier that can identify the service and the endpoint providing the service, it is possible to secure the endpoint of the current service during future communication.

### 4.5. RequestTicket Phase

**Figure 5:**
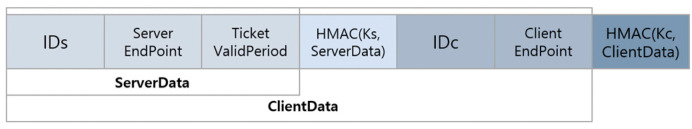
Data configured when a client needs to request a ticket from an AS.

If a client receives an OfferService message transmitted by the server and wants to use the service, check if the ticket flag is set in the message first, and if not, communicate in an existing way. If the ticket is needed, a RequestTicket message is transmitted to an AS as formatted in Figure 5. For a detailed description of notations, refer to Table 4.

In the RequestTicket message, the client configures ClientData by adding IDc, which is designated SOME/IP client ID, and ClientEndPoint after ServerData and its HMAC. Then, the client computes HMAC (Kc, ClientData). The message created in this way sets the message type to a value representing a RequestTicket message and transmits it to the AS as a SOME/IP Request message.

### 4.6. ReturnTicket Phase

When an AS receives the RequestTicket message, the following occurs:

(1) The AS finds the IDc from the transmitted packet in ClientData and a long-term key Kc corresponding to that IDc in the AS database. The AS uses Kc to calculate the HMAC value of ClientData and then compares the HMAC with the HMAC of the transmitted packet, which the client calculated. If the two HMACs do not match, a “client verification fail” error code is set, and the operation stops; if they match, the process proceeds to (2).

(2) The AS finds the IDs, which is a unique identifier for a service instance, in ServerData and a long-term key Ks corresponding to that IDs in the AS database and calculates the HMAC value of ServerData with Ks. Then, the AS compares that HMAC with the HMAC of the transmitted packet, which the server calculated. If the two HMACs do not match, a “server verification fail” error code is set, and the operation stops; if they match, the process proceeds to (3).

(3) Based on the IDs and the IDc, policy violations between nodes are checked in the AS policy data. For example, in a relationship where the service cannot be provided or is not provided, a “policy denied” error code is set in the validation code, and the operation stops. In the case of a valid relationship, the process proceeds to (4).

(4) If there is no problem from (1) to (3), a “verification success” code is set in the validation code. 

**Figure 6:**
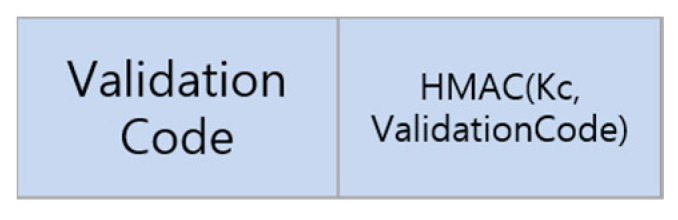
ReturnTicket structure that is configured when a validation code has an error.

**Figure 7:**
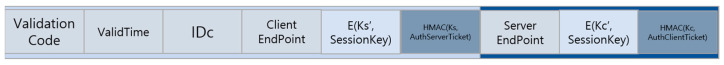
ReturnTicket structure that is configured for a successfully generated ticket from an AS.

If the validation code in Section 4.6 contains an error, the AS generates an HMAC value relating to the error code with the client’s long-term key Kc and sends it to the client as a ReturnTicket message, as shown in Figure 6. If the validation code is “verification success”, the AS is formatted as shown in Figure 7. For a detailed description of notation, refer to Table 5. The session key is generated as a cryptographically secure random value after confirming the kind of service in the AS. The overall configuration of the ReturnTicket message is divided mainly into AuthServerTicket, which is used for verification in the server, and AuthClientTicket, which is used for verification in the client.

When the validity code is “verification success”, the AS creates a ReturnTicket message that becomes formatted as shown in Figure 7, sets it as the type of the ReturnTicket message, and sends it to the client as a SOME/IP Response message. In the ReturnTicket message, a session key between the client and the server is distributed by the AS. Since the session key is encrypted with a key derived from the long-term key of each node and then transmitted, only the owner can obtain the session key. The session key is used to verify freshness to prevent a replay attack and can also be used in future studies to evaluate the confidentiality of messages. Details relating to the replay attack will be discussed later.

### 4.7. Request Phase

**Figure 8:**
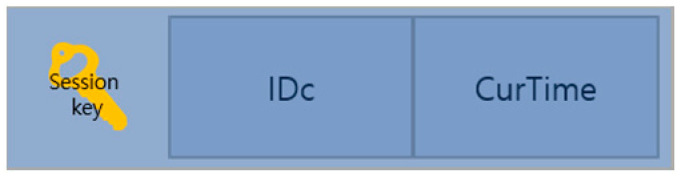
FreshnessToken format.

**Figure 9:**
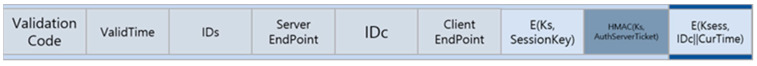
Request or SubscribeEventGroup structure that a client configures to pass AuthServerTicket and FreshnessToken to a server.

After the client receives the ReturnTicket message delivered from the AS, it first checks the HMAC part corresponding to AuthClientTicket using its key to check whether the message has been falsified or altered. If there is no problem, the AS confirms the validity code to verify the authentication and mutual relationship between the server and itself through ValidationCode in AuthServerTicket. If this is successful, the AS gets the session key by decrypting E (Kc’, SessionKey) with key Kc’ derived from its long-term key Kc. After this, FreshnessToken is created, as shown in Figure 8. FreshnessToken is encrypted with the session key after attaching IDc and CurTime, which is the time when FreshnessToken was created. This FreshnessToken is added after AuthServerTicket’s HMAC and is sent to ServerEndPoint, as shown in Figure 9.

The server receiving this message calculates the HMAC value of AuthServerTicket with its key to check message freshness. The server decrypts FreshnessToken with the acquired session key and checks whether the message was created and transmitted within the valid time using CurTime. If the transmitted time is valid, the IDc in Freshness is checked to see if it matches the IDc in the ticket. If it matches, the server sends a Response message to the client endpoint in the ticket or registers the client endpoint as a service subscriber (SubscribeEventGroupAck).

## 5. Security Analysis

### 5.1. Considering the Replay Attack

A session key distribution and freshness verification using the key are added because the server uses the client’s endpoint listed in the ticket after checking the validity between nodes based on AuthServerTicket. Suppose a malicious user stole a valid authentication ticket and repeatedly sent messages including the ticket to the server. In that case, the server would continue to send a Response message to a victim client because it is a valid ticket, and a distributed denial of service (DDoS) attack could occur in the client node. Therefore, before the server sends a response, the logic that verifies whether a legitimate ticket has been delivered is added to prevent a ticket replay attack. Also, since FreshnessToken includes the current time, it can limit the valid time of the used session key and ticket compared to the valid time in the authentication ticket. This is a one-way authentication method using a challenge–response approach, where the prover and verifier utilize a pre-shared symmetric key to verify an unforgeable timestamp.

Through this method, our suggested scheme can prevent manipulation of the endpoint, which provides and subscribes to services and is at the heart of SOME/IP MITM attacks. Thus, even if ticket freshness is guaranteed, replay attacks on tickets are rendered impossible.

### 5.2. Benefits and Concerns of Using an AS

To ensure secure communication between IoT devices, the use of an Authentication Server is often proposed as a method [21]. The proposed method also adopts a way of using an AS. There are well-known concerns about having two additional messages that need to be sent and received using an AS. Before looking into these concerns, we explain the following advantages first:

(1) As the operation arising from the extension of SOME/IP security functions is shared in the AS, operational overhead on the end nodes is relatively small, even when the node on the ECU has limited resources.

(2) Since key and policy management are performed in the AS, when a node is changed (e.g., node addition/modification/deletion), only the AS needs to be updated for the related nodes except for the node that changed. Therefore, using an AS is advantageous in terms of scalability.

With these two advantages, we adopt the model using the AS. The proposed method is rather similar to current in-vehicle architectures (not only to the central processing architecture used by Tesla and Waymo but also to the Zonal architecture that traditional automotive OEMs see as next-generation vehicle architecture). These structures make it possible to perform SW updates for segregated security functionality quickly and easily through the network. Through this, using the AS, it is easy to introduce nodes that provide new functions and advantageous for vehicle security because the area to be protected is clear. However, since a large volume of data needs to be processed at one point, a critical problem such as a single point of failure (SPOF) could occur. As such, this process requires high computing performance and high-speed communication technology. In this regard, manufacturers should design and test the system to be widely available and fault-tolerant. It also should incorporate redundant systems or fail-safe mechanisms to ensure that if one component or system were to fail, there would be a backup to prevent a catastrophic failure. Since the advantages and disadvantages of the introduced vehicle architecture are nearly identical to those proposed in this paper for the security expansion of SOME/IP, we expect that the methods offered as a supplement to address the concerns caused by the overall architecture of the vehicle can mitigate our method’s drawbacks.

### 5.3. Formal Verification

To analyze our method’s security properties, we used ProVerif, an automated tool for formally verifying security protocols, as it is designed to help security researchers identify and eliminate security vulnerabilities in cryptographic protocols. Security properties are typically secrecy, authenticity, and integrity. For these properties, the tool automatically generates attack scenarios that expose weaknesses in the protocol. As it already supports a variety of cryptographic primitives and protocol specifications, we were able to analyze our protocol easily and quickly. For details about ProVerif, refer to article [22], and note that the manual can be found in [22,23] and also online at [24].

We defined three processes for the execution processes of the server, the client, and the AS along with eight events for sending, identifying, and verifying each node following the secure scheme shown in Figure 3. The eight events are:

Events:

- event offer_service: This event denotes that a server sent an OfferService message with its HMAC to a client. It is set after the OfferService message has been sent from a server to a client.
- event request_ticket: This event denotes that a client sent a RequestTicket message with their HMAC to the AS. It is set after the RequestTicket message has been sent from a client to an AS.
- event verify_node: This event confirms the validity of a target node. It is set after validating a node’s HMAC and policies.
- event return_ticket: This event denotes that an AS sent a ReturnTicket message that includes server and client tokens as well as encrypted session key containers via their keys.
- event verify_ticket: This event confirms the validity of a node’s authentication ticket and obtains a session key. It is set after the ticket sent from an AS is validated and decrypted and the session key is obtained.
- event request: This event denotes that a client sent a Request message that includes FreshnessToken and AuthServerTicket to the server’s endpoint, configured in AuthClientTicket. It is set after a Request message is sent from a client to a server.
- event server_verify_freshness: This event confirms the validity of FreshnessToken from a client’s Request message. It is set after FreshnessToken is validated using a session key.
- event response: This event denotes that a server sent a Response message to a client. It is set after a Response message has been sent from a server to a client.

**Figure 10:**
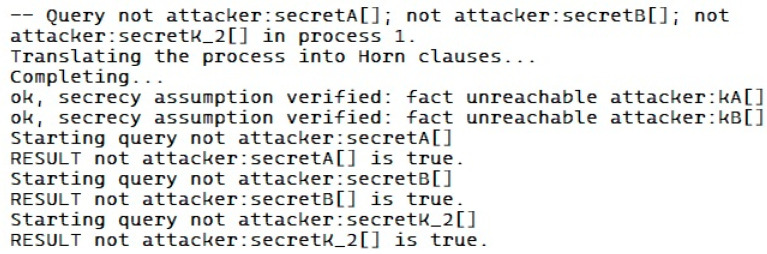
The result of security analysis via ProVerif.

We also set eight queries to confirm the security properties of the proposed security protocol. Figure 10 shows the result of the secrecy analysis of keys used for this scheme for an attacker.

Queries:

- Query not attacker:secretA[]: This query investigates whether or not a pre-defined server key shared with an AS has been leaked by an attacker. In Figure 10, it returned true, which means the key is secure.
- Query not attacker:secretB[]: This query investigates whether or not a pre-defined client key shared with an AS has been leaked by an attacker. In Figure 10, it returned true, which means the key is secure.
- Query not attacker:secretK_2[]: This query investigates whether or not a session key distributed by an AS has been leaked or not. In Figure 10, it returned true, which means the key is secure from an attacker.
- Query evinj:verify_node(x) ==> evinj:verify_node(y): This query investigates whether or not client verification occurred before server verification occurred. It returned true, which means a server node verification was always performed after a client node verification. Combined with the following query, the return_ticket event should occur after verifying both nodes.
- Query evinj:return_ticket(x,y) ==> evinj:verify_node(x): This query investigates whether or not server verification occurred before the return_ticket event occurred. It returned true, which means the return_ticket event was always sent after server node verification.
- Query evinj:request(x) ==> evinj:verify_ticket(y): This query investigates whether or not a client ticket verification occurred before the request event occurred. It returned true, which means the request event always happened after client ticket verification.
- Query evinj: verify_ticket(y) ==> evinj:verify_freshness(x): This query investigates whether or not a ticket freshness verification occurred before a server ticket verification. It returned true, which means the verify_ticket event for the server always happened after freshness verification.
- Query evinj: response(x) ==> evinj:verify_ticket(y): This query investigates whether or not a server ticket verification occurred before a response event. It returned true, which means the response event always happened after server ticket verification.

Following the listed queries, an attacker cannot modify an endpoint because the endpoint is included in each authentication ticket from an AS, and the messages are sent after verification of the tickets. By doing this, MITM and spoofing attacks are mitigated. As part of a suggested process, a client may encrypt FreshnessToken with a shared key via an AS, which ensures that the ticket’s freshness is verified before sending a response from the server. This provides mitigation of replay and DDoS attacks. To sum up, the result of ProVerif shows that our suggested security scheme has the secrecy of a session key, can authenticate nodes, and mitigates various attacks. Please refer to Appendix A for the detailed result of running ProVerif.

## 6. Evaluation

**Figure 11:**
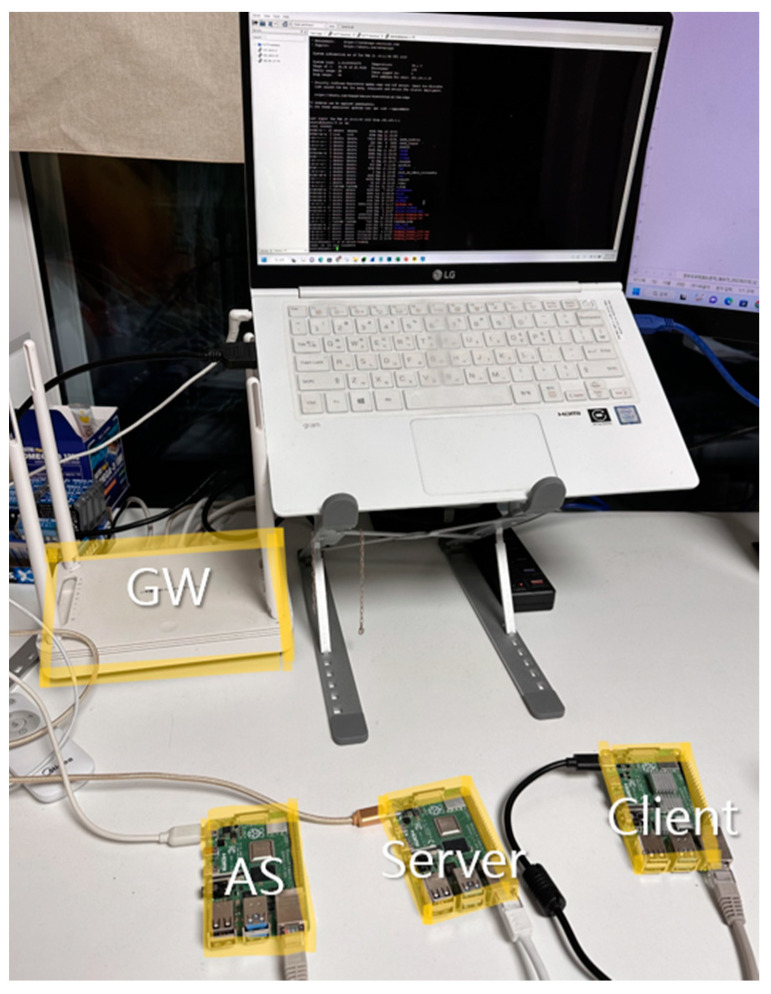
Experimental environment.

Verification was carried out through three Raspberry Pi 4 Model b+ units and one gateway with Ubuntu loaded, as shown in Figure 11, for the configuration of the server, client, and AS. We used GENEVI’s SOME/IP open source, vsomeip [25], for SOME/IP security extension implementation and compared it to another solution that called secure-vsomeip (implementation of Secure SOME/IP), which is available on GitHub. For a fair comparison, we tested on the same devices, and in the case of a symmetric algorithm, both solutions were set to operate with the AES-GCM-128 algorithm using OpenSSL. The testing security level of secure-vsomeip was set to “authentication” to provide a security level similar to that of our proposed model.

**Figure 12:**
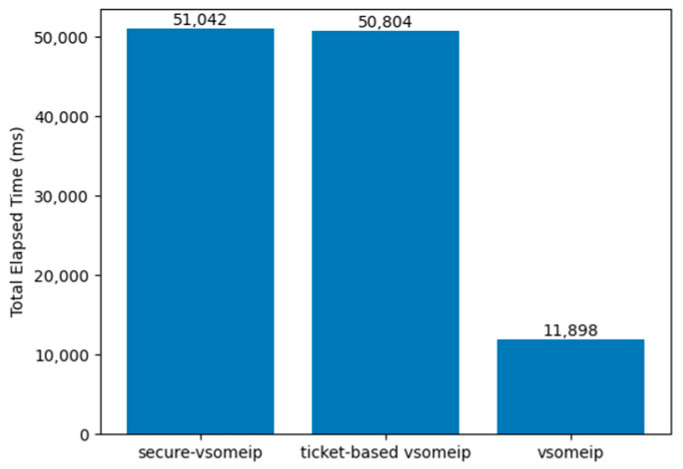
Total elapsed time to obtain a response from a server, including each security extension operation.

For convenience, we refer to our implementation as “ticket-based vsomeip”. Figure 12 shows the total time taken to send a Request message to a specific service and receive a Response message after one client app starts to run. The result represents the average value of 100 tests. In the case of secure-vsomeip, we measured the time to run the request_response client app in its benchmark and receive an actual response. While the client node was running, it also performed an additional asymmetric key operation to handshake with the server node. Ticket-based vsomeip performed symmetric key operations and included additional communication overhead with the AS. The result shows that the ticket-based vsomeip performed slightly faster than or similarly to secure-vsomeip. Please note that these measurement data represent the total Round Trip Time (RTT) and include the time required for the operations on all nodes. CPU usage refers to how many resources the processor consumes to perform a given task. Therefore, we proceeded to measure the CPU usage as well. When measuring the CPU on the server side, using secure-vsomeip, we observed an average value of 25.546%. In comparison, ticket-based vsomeip showed a usage of 6.67%, indicating a reduction of approximately 76%. This difference in CPU usage can be attributed to the way each method processes the task and the complexity of the algorithms involved.

**Figure 13:**
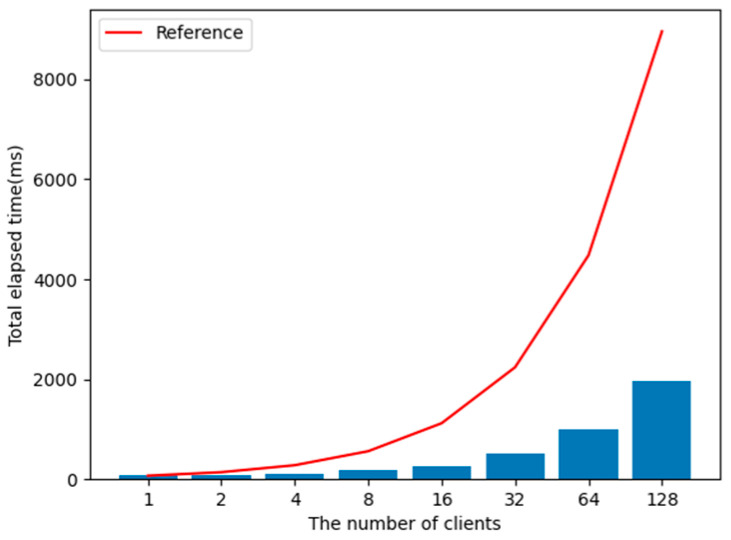
Evaluated elapsed time to handle all RequestTicket messages on an AS.

Since this proposed method performs most of the core operations for security expansion in an AS, it reduces overhead caused by added security implementations on the server and the client. Since ticket requests from multiple nodes can be demanded simultaneously, it is necessary to verify the processing capacity of the AS according to the increase in client ticket requests. Figure 13 illustrates the average measurements corresponding to the number of clients over 100 tests.

All clients receive an OfferService message that requires a ticket provided by the server and want to use the service, then send RequestTicket messages to the AS. The measured time is the total time taken by the AS to transmit ReturnTicket messages to all clients (see Figure 13, which shows a linear increase in the elapsed time in relation to increasing numbers of clients). Due to parallel processing, the actual measured results show a speed increase that is less than the anticipated linear growth based on increased client nodes. As such, using an AS is a feasible method.

## 7. Conclusions

As the automotive industry continues to evolve and the demand for data increases, Ethernet technology is poised to play an increasingly important role in vehicle communication. In this context, the security of SOME/IP, an Ethernet standard protocol, is crucial for ensuring safety, privacy, and cybersecurity in modern vehicles. The proposed method mitigates known MITM attacks on SOME/IP using a ticket that guarantees end node endpoints from an AS. Compared to methods proposed in other studies, our approach offers an advantage in terms of scalability, requiring only minimal additional work to accommodate changes such as adding or deleting nodes. This is thanks to the fact that key changes and policy changes due to node changes are possible only with an AS software update. Additionally, the proposed method can be implemented and used without significantly affecting the existing SOME/IP protocol, making it compatible with current systems. Furthermore, as it relies only on symmetric key operations, the operation of our method is relatively light. Through the session key distributed by the AS and FreshnessToken protected by the key, the server can self-verify the freshness of a ticket, effectively preventing replay attacks. We proved the security properties of the proposed method through ProVerif and confirmed feasibility in our evaluation. In future research, we plan to consider the use of a distributed session key to ensure message confidentiality based on the importance of the service.

## Figures and Tables

**Table 1:** Comparison table showing various schemes.

Scheme	Scalability	Granularity	Overhead	Compatibility	Efficiency
Ticket-based SOME/IP	High	Fine-grained	Low	Supported	High
Secure SOME/IP	Low	Fine-grained	High	Supported	Low
SESO-RC	Low	Coarse grained	High	Not Supported	High
SESO-AS	High	Coarse grained	Low	Not Supported	Low

**Table 2:** Notations for the proposed scheme’s overall flow in Figure 3.

Notation	Description
OfferService	Original OfferService payload
Request	Original Request payload
SD	ServerData that is configured by a server
Ks	Pre-configured server’s symmetric key shared with an AS
Kc	Pre-configured client’s symmetric key shared with an AS
H (key, value)	HMAC calculated value for ‘value’ by ‘key’
CD	ClientData that is configured by a client
AST	AuthServerTicket that is configured by an AS
ACT	AuthClientTicket that is configured by an AS
FT	FreshnessToken that is configured by a client

**Table 3:** Descriptions of notations for the refined OfferService message data in Figure 4.

Notation	Description
OfferService	Original OfferService payload
IDs	An identifier that can uniquely identify a service instance (ServiceID||InstanceID)
ServerEndPoint	IP address and port number of the service’s current server
TicketValidPeriod	Period during which the issued ticket is valid
ServerData	Data from IDs to TicketValidPeriod
HMAC (Ks, ServerData)	HMAC value for ServerData made with a long-term server key

**Table 4:** Descriptions of notation for RequestTicket message data in Figure 5.

Notation	Description
ServerData	ServerData received from a server through OfferService
HMAC (Ks, ServerData)	HMAC value for ServerData received from a server through OfferService
IDc	Identifier that can uniquely identify a client (ClientID)
ClientEndPoint	Client’s current IP address and port number
ClientData	Data from ServerData to ClientEndPoint
HMAC (Kc, ClientData)	HMAC value for ClientData made with a long-term client key

**Table 5:** Descriptions of notations for ReturnTicket message data in Figure 7.

Notation	Description
ValidationCode	Validity result code checked by an AS in Section 4.5, such as peer authority and policy checks
ValidTime	Time taken for the AS to perform ticket issuance plus the TicketValidPeriod delivered by the server in OfferService
IDc	Client ID
ClientEndPoint	Client’s current IP address and port number
E(Ks’, SessionKey)	Encrypted session key with the derived server encryption key
AuthServerTicket	Data from ValidationCode to E (Ks’, SessionKey)
HMAC (Ks, AuthServerTicket)	HMAC value of AuthServerTicket generated with a long-term server key
ServerEndPoint	Server’s IP address and port number
E (Kc’, SessionKey)	Encrypted session key with the derived client encryption key
AuthClientTicket	Data from AuthServerTicket to E (Kc’, SessionKey)
HMAC (Kc, AuthClientTicket)	HMAC value of AuthClientTicket generated with a long-term client key

## Data Availability

There is no additional data.

## Appendix A

The result of running the ProVerif

Process 0 (that is, the initial process):

{1}new kA;

{2}new kB;

{3}let hostA = host(kA) in

{4}let hostB = host(kB) in

{5}out(c, hostA);

{6}out(c, hostB);

(

       {7}!

       {8}new SD;

       {9}event offer_service(hostB);

       {10}out(c, (hostA,hostB,SD,keyhash((hostA,hostB,SD),kA)));

       {11}event offer_service(hostB);

       {12}in(c, (=hostA,hostB1,Ea1,h31,ft));

       {13}if (h31 = keyhash((hostA,hostB1,Ea1),kA)) then

       {14}event verify_ticket(hostA);

       {15}let (=hostA,=hostB,secretK) = decrypt(Ea1,kB) in

       {16}let (=hostB,t2) = decrypt(ft,secretK) in

       {17}event verify_freshness(hostB1);

       {18}event response(hostB1)

) | (

       {19}!

       {20}new CD;

       {21}in(c, (hostA1,=hostB,SD1,h1));

       {22}out(c, (hostA1,hostB,SD1,h1,CD,keyhash((hostA1,hostB,SD1,h1,CD),kB)));

       {23}event request_ticket();

       {24}in(c, (hostA2,=hostB,Ea,h3,Eb1,h4));

       {25}if (h4 = keyhash((hostA2,hostB,Ea,h3,Eb1),getkey(hostB))) then

       {26}let (=hostB,=hostA,secretK_1) = decrypt(Eb1,kB) in

       {27}new t1;

       {28}event verify_ticket(hostB);

       {29}out(c, (hostA2,hostB,Ea,h3,encrypt((hostB,t1),secretK_1)));

       {30}event request(hostA2)

) | (

       {31}!

       {32}in(c, (hostA2_1,hostB1_1,SD12,h12,CD12,h22));

       {33}if (h22 = keyhash((hostA2_1,hostB1_1,SD12,h12,CD12),getkey(hostB))) then

       {34}event verify_node(hostB);

       {35}if (h12 = keyhash((hostA2_1,hostB1_1,SD12),getkey(hostA))) then

       {36}event verify_node(hostA);

       {37}new k;

    {38}out(c, (hostA2_1,hostB1_1,encrypt((hostA2_1,hostB1_1,k),getkey(hostA)),keyhash((encrypt((hostA2_1,hostB1_1,k),getkey(hostA))),getkey(hostA)),encrypt((hostB1_1,hostA2_1,k),getkey(hostB)),keyhash((encrypt((hostA2_1,hostB1_1,k),getkey(hostA)),keyhash((encrypt((hostA2_1,hostB1_1,k),getkey(hostA))),getkey(hostA)),encrypt((hostB1_1,hostA2_1,k),getkey(hostB))),getkey(hostB))));

       {39}event response_ticket(hostA2_1,hostB1_1)

)

-- Process 1 (that is, process 0, with let moved downwards):

{1}new kA;

{2}new kB;

{3}let hostA = host(kA) in

{5}out(c, hostA);

{4}let hostB = host(kB) in

{6}out(c, hostB);

(

       {7}!

       {8}new SD;

       {9}event offer_service(hostB);

       {10}out(c, (hostA,hostB,SD,keyhash((hostA,hostB,SD),kA)));

       {11}event offer_service(hostB);

       {12}in(c, (=hostA,hostB1,Ea1,h31,ft));

       {13}if (h31 = keyhash((hostA,hostB1,Ea1),kA)) then

       {14}event verify_ticket(hostA);

       {15}let (=hostA,=hostB,secretK) = decrypt(Ea1,kB) in

       {16}let (=hostB,t2) = decrypt(ft,secretK) in

       {17}event verify_freshness(hostB1);

       {18}event response(hostB1)

) | (

       {19}!

       {20}new CD;

       {21}in(c, (hostA1,=hostB,SD1,h1));

       {22}out(c, (hostA1,hostB,SD1,h1,CD,keyhash((hostA1,hostB,SD1,h1,CD),kB)));

       {23}event request_ticket();

       {24}in(c, (hostA2,=hostB,Ea,h3,Eb1,h4));

       {25}if (h4 = keyhash((hostA2,hostB,Ea,h3,Eb1),getkey(hostB))) then

       {26}let (=hostB,=hostA,secretK_1) = decrypt(Eb1,kB) in

       {27}new t1;

       {28}event verify_ticket(hostB);

       {29}out(c, (hostA2,hostB,Ea,h3,encrypt((hostB,t1),secretK_1)));

       {30}event request(hostA2)

) | (

       {31}!

       {32}in(c, (hostA2_1,hostB1_1,SD12,h12,CD12,h22));

       {33}if (h22 = keyhash((hostA2_1,hostB1_1,SD12,h12,CD12),getkey(hostB))) then

       {34}event verify_node(hostB);

       {35}if (h12 = keyhash((hostA2_1,hostB1_1,SD12),getkey(hostA))) then

       {36}event verify_node(hostA);

       {37}new k;

    {38}out(c, (hostA2_1,hostB1_1,encrypt((hostA2_1,hostB1_1,k),getkey(hostA)),keyhash((encrypt((hostA2_1,hostB1_1,k),getkey(hostA))),getkey(hostA)),encrypt((hostB1_1,hostA2_1,k),getkey(hostB)),keyhash((encrypt((hostA2_1,hostB1_1,k),getkey(hostA)),keyhash((encrypt((hostA2_1,hostB1_1,k),getkey(hostA))),getkey(hostA)),encrypt((hostB1_1,hostA2_1,k),getkey(hostB))),getkey(hostB))));

       {39}event response_ticket(hostA2_1,hostB1_1)

)

-- Query not attacker:secretA[]; not attacker:secretB[]; not attacker:secretK_2[] in process 1.

Translating the process into Horn clauses...

Completing...

ok, secrecy assumption verified: fact unreachable attacker:kA[]

ok, secrecy assumption verified: fact unreachable attacker:kB[]

Starting query not attacker:secretA[]

RESULT not attacker:secretA[] is true.

Starting query not attacker:secretB[]

RESULT not attacker:secretB[] is true.

Starting query not attacker:secretK_2[]

RESULT not attacker:secretK_2[] is true.

-- Query evinj:verify_node(x) ==> evinj:verify_node(y) in process 1.

Translating the process into Horn clauses...

Completing...

ok, secrecy assumption verified: fact unreachable attacker:kA[]

ok, secrecy assumption verified: fact unreachable attacker:kB[]

Starting query evinj:verify_node(x) ==> evinj:verify_node(y)

goal reachable: begin:verify_node(host(kB[])),@occ34_1 -> end:@occ36_1,verify_node(host(kA[]))

The hypothesis occurs strictly before the conclusion.

Abbreviations:

SD_1 = SD[!1 = @sid]

CD_1 = CD[!1 = @sid_1]

@occ36_1 = @occ36[h22 = keyhash((host(kA[]),host(kB[]),SD_1,keyhash((host(kA[]),host(kB[]),SD_1),kA[]),CD_1),kB[]),CD12 = CD_1,h12 = keyhash((host(kA[]),host(kB[]),SD_1),kA[]),SD12 = SD_1,hostB1_1 = host(kB[]),hostA2_1 = host(kA[]),!1 = @sid_2]

@occ34_1 = @occ34[h22 = keyhash((host(kA[]),host(kB[]),SD_1,keyhash((host(kA[]),host(kB[]),SD_1),kA[]),CD_1),kB[]),CD12 = CD_1,h12 = keyhash((host(kA[]),host(kB[]),SD_1),kA[]),SD12 = SD_1,hostB1_1 = host(kB[]),hostA2_1 = host(kA[]),!1 = @sid_2]

goal reachable: attacker:hostA2_2 & attacker:SD12_1 & attacker:h12_1 -> end:@occ34_1,verify_node(host(kB[]))

The 1st, 2nd, 3rd hypotheses occur before the conclusion.

Abbreviations:

CD_1 = CD[!1 = @sid]

@occ34_1 = @occ34[h22 = keyhash((hostA2_2,host(kB[]),SD12_1,h12_1,CD_1),kB[]),CD12 = CD_1,h12 = h12_1,SD12 = SD12_1,hostB1_1 = host(kB[]),hostA2_1 = hostA2_2,!1 = @sid_1]

RESULT evinj:verify_node(x) ==> evinj:verify_node(y) is true.

-- Query evinj:response_ticket(x,y) ==> evinj:verify_node(x) in process 1.

Translating the process into Horn clauses...

Completing...

ok, secrecy assumption verified: fact unreachable attacker:kA[]

ok, secrecy assumption verified: fact unreachable attacker:kB[]

Starting query evinj:response_ticket(x,y) ==> evinj:verify_node(x)

goal reachable: begin:verify_node(host(kA[])),@occ36_2 & begin:verify_node(host(kB[])),@occ34_2 -> end:@occ39_1,response_ticket(host(kA[]),host(kB[]))

The 1st, 2nd hypotheses occur strictly before the conclusion.

Abbreviations:

SD_1 = SD[!1 = @sid]

CD_1 = CD[!1 = @sid_1]

@occ39_1 = @occ39[h22 = keyhash((host(kA[]),host(kB[]),SD_1,keyhash((host(kA[]),host(kB[]),SD_1),kA[]),CD_1),kB[]),CD12 = CD_1,h12 = keyhash((host(kA[]),host(kB[]),SD_1),kA[]),SD12 = SD_1,hostB1_1 = host(kB[]),hostA2_1 = host(kA[]),!1 = @sid_2]

@occ36_2 = @occ36_1[h22 = keyhash((host(kA[]),host(kB[]),SD_1,keyhash((host(kA[]),host(kB[]),SD_1),kA[]),CD_1),kB[]),CD12 = CD_1,h12 = keyhash((host(kA[]),host(kB[]),SD_1),kA[]),SD12 = SD_1,hostB1_1 = host(kB[]),hostA2_1 = host(kA[]),!1 = @sid_2]

@occ34_2 = @occ34_1[h22 = keyhash((host(kA[]),host(kB[]),SD_1,keyhash((host(kA[]),host(kB[]),SD_1),kA[]),CD_1),kB[]),CD12 = CD_1,h12 = keyhash((host(kA[]),host(kB[]),SD_1),kA[]),SD12 = SD_1,hostB1_1 = host(kB[]),hostA2_1 = host(kA[]),!1 = @sid_2]

RESULT evinj:response_ticket(x,y) ==> evinj:verify_node(x) is true.

-- Query evinj:request(x) ==> evinj:verify_ticket(x) in process 1.

Translating the process into Horn clauses...

Completing...

ok, secrecy assumption verified: fact unreachable attacker:kA[]

ok, secrecy assumption verified: fact unreachable attacker:kB[]

Starting query evinj:request(x) ==> evinj:verify_ticket(x)

RESULT evinj:request(x) ==> evinj:verify_ticket(x) is true.

-- Query evinj:response(x) ==> evinj:verify_freshness(x) in process 1.

Translating the process into Horn clauses...

Completing...

ok, secrecy assumption verified: fact unreachable attacker:kA[]

ok, secrecy assumption verified: fact unreachable attacker:kB[]

Starting query evinj:response(x) ==> evinj:verify_freshness(x)

RESULT evinj:response(x) ==> evinj:verify_freshness(x) is true.
